# Safety of Credelio Quattro™ (lotilaner, moxidectin, praziquantel, and pyrantel chewable tablets) in homozygous MDR1-mutant collie dogs

**DOI:** 10.1186/s13071-025-06795-y

**Published:** 2025-04-23

**Authors:** Kari L. Riggs, Xinshuo Wang, Scott Wiseman

**Affiliations:** 1 Elanco Animal Health, 2500 Innovation Way, Greenfield, IN 46140 USA; 2 Elanco Animal Health, Form 2, Bartley Way, Bartley Wood Business Park, Hook, RG27 9XA UK

**Keywords:** Credelio Quattro, Lotilaner, Moxidectin, Praziquantel, Pyrantel, Safety, Collie, MDR1

## Abstract

**Background:**

Multidrug resistance-1 (MDR1) mutant dogs have diminished or lack P-glycoprotein (Pgp) expression at the blood–brain barrier and are therefore more susceptible to neurotoxicity caused by macrocyclic lactones and other Pgp substrates due to increased drug penetration into the brain. Therefore, the safety of products containing macrocyclic lactones are required to be evaluated in this sensitive population. Credelio Quattro (lotilaner, moxidectin, praziquantel, and pyrantel chewable tablets) is a novel endectocide for monthly oral administration in dogs. As moxidectin is a macrocyclic lactone, Credelio Quattro was administered to homozygous MDR1 mutant Collie dogs to evaluate the safety of the product.

**Methods:**

The study employed a completely randomized and blinded design, where dogs were allocated to one of four treatment groups. A total of 32 dogs were divided into 4 groups (placebo control, 1×, 2×, or 5×, the maximum recommended labeled dose of Credelio Quattro) each consisting of 8 dogs. Treatment was administered on 3 consecutive occasions, 28 days apart. Dogs were evaluated pre-dose and through 72-h post-treatment using the avermectin sensitive (AVS) categories of seizures or convulsions, ataxia, depression, mydriasis, muscle tremors, and salivation/drooling/vomiting. The assessment of safety was based on AVS scores, general health observations, body weight, and physical examinations.

**Results:**

Credelio Quattro was well tolerated with no serious adverse events. There were no incidents of seizures or convulsions, ataxia, mydriasis, or muscle tremors observed. Salivation/drooling/vomiting was the most frequent observation, occurring in all groups, and most frequently in the 5× group. Vomiting is a dose-dependent effect observed for Credelio Quattro in healthy dogs and is therefore unlikely to represent a neurological effect in MDR1 dogs. Depression was observed in one dog in each of the 0×, 2×, and 5× groups. This was likely a spurious result versus true toxicity, as the sign was subtle and occurred singularly including at 0×. For all AVS signs, the events were transient, and dogs recovered without any intervention.

**Conclusions:**

Credelio Quattro was well tolerated and is safe in MDR1 mutant dogs up to 5× the maximum recommended dose following three consecutive monthly administrations.

**Graphical Abstract:**

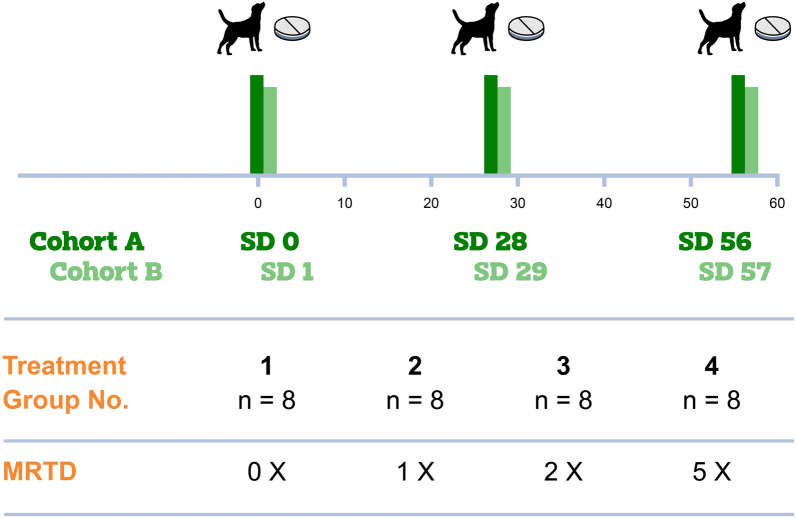

## Background

Multidrug resistance-1 (MDR1) mutant dogs have a genetic defect in their ATP-binding cassette, subfamily B member 1 (ABCB1) gene. The ABCB1 gene encodes P-glycoprotein (Pgp) which plays a crucial role in the absorption and excretion of molecules and the blood–brain barrier function in dogs. Pgp can affect processing of orally administered molecules by hindering their absorption in the intestines and increasing their elimination through the kidneys and liver [1–3]. Thus, mutations in the ABCB1 gene lead to a truncated non-functional Pgp [4], which in turn alters the pharmacokinetic properties of Pgp transported drugs, leading to an increase in drug bioavailability and greater systemic drug concentrations, with accumulation of drug in the central nervous system (CNS) [5, 6].

The blood–brain barrier is a protective barrier that separates the blood circulation from the brain and spinal cord, preventing the entry of Pgp substrates into the central nervous system (CNS). Certain Pgp substrates, such as macrocyclic lactones (e.g., ivermectin, moxidectin, doramectin), loperamide, digoxin, acepromazine, ondansetron, vincristine, vinblastine, doxorubicin, and apomorphine, can have toxic effects in dogs with MDR1 mutations due to lack of functional Pgp resulting in accumulation in the CNS [7, 8]. Dogs with two copies of the mutated MDR1 gene (homozygous MDR1 −/−) tend to experience more severe symptoms of drug neurotoxicity due to higher drug accumulation compared with dogs with one copy of the mutation (heterozygous MDR1 +/-) [9, 10]. The MDR1 mutation has been found in many mixed-breed dogs and in pure bred Australian Shepherds (standard and miniature), Border Collie, Collie, English Shepherd, German Shepherd, Longhaired Whippet, Old English Sheepdog, Shetland Sheepdog, and Silken Windhound [11, 12].

Macrocyclic lactones (ML), including avermectins and milbemycins, are a group of compounds that originate from soil-dwelling fungi known as Streptomyces. These compounds possess strong anthelmintic and ectoparasitic properties, making them extensively utilized in veterinary medicines to combat parasitic diseases [13]. In vertebrates, the mechanism-based toxicity of all MLs is attributed to their binding to neuronal GABA-gated chloride channels. As a result, MDR1 mutant dogs, which lack Pgp expression at the blood–brain barrier, are particularly susceptible to neurotoxicity due to increased penetration into the brain by a number of substances including MLs. While recent studies have validated that the administration of MLs to MDR1 mutant dogs may not necessarily lead to neurological symptoms, the level of safety is greatly influenced by factors such as the specific dosage, route of application, frequency of administration, potential interaction (competition) at PgP level with other PgP substrates, and individual compound utilized [4]. The ivermectin-sensitive phenotype was first reported in Collies in 1980, in which life-threatening toxicity occurred due to accumulation of ivermectin in the CNS [7], which was later attributed to the mutation of the MDR1 gene [9]. For this reason, it is required to test ML containing preventives in this sensitive population.

Credelio Quattro is a novel chewable tablet containing moxidectin (a ML) along with anthelmintics, praziquantel and pyrantel, and ectoparasiticide lotilaner. It was developed to provide broad-spectrum efficacy against most endo- and ecto-parasites in dogs, when administered monthly. This broad-spectrum chewable tablet intends to improve compliance by preventing/treating common parasites in a single tablet versus the multiple medications currently required to achieve the same spectrum of activity. The study presented in this publication evaluated the safety of Credelio Quattro when administered orally to avermectin-sensitive Collies (MDR1 −/−) at 0×, 1×, 2×, and 5× doses for three consecutive occasions, every 28 days.

## Methods

The test facility Institutional Animal Care and Use Committee approved the use of animals and all animal procedures. The study was conducted in general accordance with (a) applicable regulations of the United States Food and Drug Administration (FDA) Good Laboratory Practice (GLP) Regulations for nonclinical laboratory studies Standards, 21 CFR Part 58 (5 October 1987) [14]; (b) study protocol, and Cheri-Hill Kennel & Supply, Inc. standard operating procedures (SOPs). The randomization and descriptive statistics were conducted at BioSTAT Consultants, Inc. (Mattawan, MI, USA); and clinical pathology assessments were performed at Antech Diagnostics GLP (Morrisville, NC, USA).

### Experimental animals

A total of 32 dogs homozygous for the MDR1 mutation were selected from a colony of known avermectin-sensitive Collies. These dogs were demonstrated to be avermectin sensitive following administration of oral ivermectin at 120 μg/kg or less (i.e., they were phenotypically sensitive).

Only dogs that had not received any lotilaner within 6 months, any MLs within 3 months (except for the ivermectin used to confirm sensitivity, which was not used within 28 days), or praziquantel or pyrantel within 1 week prior to day 0 were utilized in the study. Before being selected for study, dogs also had to pass a pretreatment physical exam and have normal clinical pathology parameters.

### Randomization and treatment

The study employed a randomized and blinded design, where the dogs were allocated to one of four treatment groups through complete randomization. Due to the number of dogs and frequency of observation for avermectin sensitivity (AVS) scoring, the 32 dogs were divided into 2 equal cohorts with 16 dogs per cohort, so that all assessments could be conducted within the specified window. The study utilized four treatment groups with each treatment group consisting of eight dogs, with four dogs per cohort per treatment group (Table 1). The randomization plan was created using SAS (SAS Institute, Cary NC; version 9.4).

**Table 1:** Study design information

Treatment group	Treatment	Target MRTD (mg/kg)	Dogs^a^
1	0× (Vehicle Control)	0	8
2	1×	Lotilaner = 40 Moxidectin = 0.04 Praziquantel = 10 Pyrantel = 10	8
3	2×	Lotilaner = 80 Moxidectin = 0.08 Praziquantel = 20 Pyrantel = 20	8
4	5×	Lotilaner = 200 Moxidectin = 0.20 Praziquantel = 50 Pyrantel = 50	8

^a^ Dogs were divided into two equal cohorts with four dogs/cohort/treatment group

According to VICH GL 43 guideline, the margin of safety is evaluated by considering multiples of the maximum recommended therapeutic dose (MRTD) [15]. The MRTD refers to the dose intended for the lightest weight dog within the broadest dose range. For Credelio Quattro, the MRTD is approximately 40 mg/kg lotilaner + 0.04 mg/kg moxidectin + 10 mg/kg praziquantel + 10 mg/kg pyrantel. In this study, group 1 was the negative control and received 0× (placebo tablet), group 2 received 1×, group 3 received 2×, and group 4 received 5× dose of Credelio Quattro (see Table 1). The dogs were dosed orally in the fed state on study day (SD) 0, 28, and 56 for cohort A, and on SD 1, 29, and 57 for cohort B. The Credelio Quattro dose for each dog was determined by their most recent body weight. A combination of whole tablets was utilized to get as close as possible to the MRTD. Dogs were never under-dosed by more than 10% of the MRTD. In cases where dosing by less than 10% of the MRTD was not achievable, the dose exceeded the maximum required amount.

### Clinical observations

Individual general health observations (GHOs) to evaluate general health were performed twice daily (AM and PM), at least 4 h apart, from SD −7 to the conclusion of the study. Additionally, body weights and physical examinations were conducted prior to each dosing cycle. To assess for signs of avermectin-associated toxicity, dogs were observed pre-dose and 1, 2, 3, 4, 5, and 6 h (± 15 min) post-treatment as well as at 8, 12, 18, and 24 h (± 30 min) and 36, 48, 60, and 72 h (± 2 h) post-treatment for avermectin sensitivity scoring. All AVS assessments were conducted by a single trained blinded observer. Table 2 provides the AVS scoring criteria utilized in this study, which was based on previously established methodology [16]. The timepoints evaluated were chosen to cover the period of acute neurological risk following peak plasma concentrations of moxidectin, as the Cmax is typically 2–3 h post oral administration [17].

**Table 2:** Signs of avermectin associated toxicity and scoring criteria

Clinical sign*	Score	Description of criteria
Depression	0: normal	Normal response to stimuli
	1: mild	Lethargy. Response to stimuli subdued. Appears confused
	2: moderate	Semi-responsive, stupor. Will respond only to persistent stimuli
	3: severe	Recumbent, weak, nonresponsive. Little or no response to stimulus, including deep pain (toe pinch). Unaware of surroundings
Ataxia	0: normal	Usual gait and posture
	1: mild	Detectable incoordination but moves with little difficulty
	2: moderate	Able to stand but moves with difficulty because of incoordination/ataxia
	3: severe	Unable to remain standing without support or unable to rise from recumbency because of incoordination/ataxia
Mydriasis**	0: normal	Usual pupillary response
	1: mild	Partial mydriasis, responds to light
	3: severe	Complete mydriasis, does not respond to light
Salivation/drooling/vomiting	0: normal	Salivation/drooling not apparent
	1: mild	Salivation/drooling minimal or 1 time vomit
	2: moderate	Consistent/moderate salivation/drooling or two times vomit
	3: severe	Excessive salivation/drooling or greater than two times vomit
Muscle tremor	0: normal	None, no apparent muscle tremors
	1: mild	Localized/intermittent muscle tremors noted
	2: moderate	Moderate whole body muscle tremors
	3: severe	Marked whole body muscle tremors

*Seizures and convulsions were documented separately as part of this evaluation

**Mydriasis score of 2 is not used for this study

### Food and water

Dogs had unrestricted access to fresh drinking water throughout the study. During acclimation and on nontreatment days, dogs were provided with unrestricted access to dry food (The Pride 21/10 Maintenance Formula; The Hyland Company Inc.; Ashland, KY). To encourage consumption of wet-canned food prior to dosing and to ensure all animals were in a similarly fed state, animals were fasted the night prior to each dosing day. Dogs in cohort A were fasted overnight prior to SD 0, 28, and 56, while dogs in cohort B were fasted overnight prior to SD 1, 29, and 57 (approximately 12 h), followed by presentation of a highly palatable wet-canned food [Pedigree (Mars Petcare US; Franklin, TN)] at approximately 25% of the manufacturer’s recommended daily amount on the basis of body weight. The consumption was assessed within a period of 20 min. If the food was not consumed within this time, the dog was hand-fed by placing small portions of food into the back of its mouth. Oral dosing occurred approximately 30–45 min post feeding. Following oral dosing, the dogs were provided with their regular portion of dry food.

### Statistical analysis

Descriptive statistical analyses were conducted for each of the AVS variable numeric scores (ataxia, depression, mydriasis, muscle tremors, and salivation/drooling/vomiting) as well as for the total score defined as the sum of the individual sensitivity scores [15]. Data were analyzed with the statistical software package SAS® (version 9.4, SAS Institute Inc., Cary, NC, USA).

## Results

### Dosing

Actual doses received were very close to the target, thus thoroughly testing the 1×, 2×, and 5× doses. A summary of the mean and standard deviation of doses received across all dose cycles is presented in Table 3.

**Table 3:** Summary of doses received across all dose cycles

Active Ingredient	Mean ± SD dose received (mg/kg)		
	1×	2×	5×
Lotilaner	39.15 ± 0.396	79.90 ± 0.412	198.49 ± 0.981
Moxidectin	0.039 ± 0.0004	0.080 ± 0.0004	0.198 ± 0.0010
Praziquantel	9.92 ± 0.100	20.24 ± 0.104	50.28 ± 0.248
Pyrantel	9.92 ± 0.100	20.24 ± 0.104	50.28 ± 0.248

### AVS scoring

There were no incidents of seizures or convulsions, ataxia, mydriasis, or muscle tremors observed in any dog in any treatment group, including the highest dose, in any dose cycle. Table 4 provides the mean and standard deviation of the AVS scores after each dose cycle and when all dose cycles are combined. Table 5 provides the total number of abnormal observations and the number of affected animals.

**Table 4:** Summary of the mean ± SD of AVS scores after each dose cycle and all dose cycles combined

	AVS clinical signs^a^	0× (control)	1×	2×	5×
First dose cycle	Depression	0.00 ± 0.00	0.00 ± 0.00	0.04 ± 0.13	0.00 ± 0.00
	Salivation/drooling/vomiting	0.01 ± 0.03	0.08 ± 0.20	0.07 ± 0.18	0.21 ± 0.27
	Overall	0.01 ± 0.03	0.08 ± 0.20	0.12 ± 0.30	0.21 ± 0.27
Second dose cycle	Depression	0.00 ± 0.00	0.00 ± 0.00	0.00 ± 0.00	0.01 ± 0.03
	Salivation/drooling/vomiting	0.01 ± 0.03	0.07 ± 0.10	0.06 ± 0.13	0.21 ± 0.24
	Overall	0.01 ± 0.03	0.07 ± 0.10	0.06 ± 0.13	0.22 ± 0.24
Third dose cycle	Depression	0.02 ± 0.05	0.00 ± 0.00	0.00 ± 0.00	0.00 ± 0.00
	Salivation/drooling/vomiting	0.01 ± 0.03	0.10 ± 0.18	0.06 ± 0.10	0.24 ± 0.29
	Overall	0.03 ± 0.05	0.10 ± 0.18	0.06 ± 0.10	0.24 ± 0.29
All cycles	Depression	0.01 ± 0.02	0.00 ± 0.00	0.01 ± 0.04	0.00 ± 0.01
	Salivation/drooling/vomiting	0.01 ± 0.02	0.08 ± 0.15	0.07 ± 0.13	0.22 ± 0.25
	Overall	0.01 ± 0.02	0.08 ± 0.15	0.08 ± 0.17	0.23 ± 0.25

^a^AVS clinical signs are only listed for those categories where dogs had abnormal signs. No signs were observed in any dog for seizures or convulsions, ataxia, mydriasis, or muscle tremors

**Table 5:** Total incidence of avermectin sensitivity: number of abnormal observations (number of affected animals)

Observations	Total incidence of avermectin sensitivity ^a^ No. of observations (no. of affected animals)											
	Cycle 1				Cycle 2				Cycle 3			
	0×	1×	2×	5×	0×	1×	2×	5×	0×	1×	2X	5×
Depression	–	–	5 (1)	–	–	–	–	1 (1)	2 (1)	–	–	–
Salivation/drooling	–	8 (1)	7 (1)	16 (4)	–	6 (2)	5 (1)	12 (3)	–	10 (2)	5 (2)	15 (2)
Vomiting	1 (1)	1 (1)	1 (1)	9 (5)	1 (1)	1 (1)	1 (1)	11 (7)	1 (1)	1 (1)	3 (3)	10 (8)
Salivation/drooling/vomiting^b^	1 (1)	9 (2)	8 (2)	21 (6)	1 (1)	7 (3)	6 (2)	18 (7)	1 (1)	11 (3)	7 (4)	23 (8)

No. number, *–* no sign observed

^a^AVS clinical signs are only listed for those categories where dogs had abnormal signs. No signs were observed in any dog for seizures or convulsions, ataxia, mydriasis, or muscle tremors

^b^Note: the salivation/drooling/vomiting row provides the incidence and number of animals experiencing salivation/drooling and/or vomiting. This may not equate to the sum of the two preceding rows as some animals experienced both signs

Results suggest an increasing dose-dependent effect of treatment on salivation/drooling/vomiting, with the 5× group having the highest score. This leads to an apparent dose dependent effect on the overall score.

The data in this study for the salivation/drooling/vomiting sign were collected by indicating whether the score was related to salivation/drooling only, vomiting only, or the combination of salivation/drooling and vomiting. As observed in Table 5, both salivation/drooling and vomiting increase with dose, with the 5× group having the highest incident and animal rates (separately and when combined into the same clinical sign). Both salivation and vomiting can be neurotoxicity signs, but they are also not specific just to neurotoxicity as they can also be signs for allergies, infections, dental issues, and gastrointestinal distress. Vomiting has been reported in normal healthy Beagle dogs at these same dose levels [18], with vomiting increasing with dose. Hypersalivation associated with vomiting was also reported in healthy Beagle dogs at the 5× dose level [18]. Therefore, vomiting and salivation associated with vomiting may or may not be related to the MDR1 mutant dogs and is not considered a true sign of neurotoxicity.

Other than salivation/drooling/vomiting, the only sign observed was depression in one dog receiving 2× in the first dose cycle, one dog receiving 5× in the second dose cycle, and one dog receiving 0× in the third dose cycle (see Table 5). As depression was only seen in one dog in each of the 0×, 2×, and 5× groups singularly in separate dose cycles, and due to the subjective nature of the scoring and subtlety of signs, these signs were likely incidental observations rather than true toxicity. For all AVS signs, the events were transient, and dogs recovered on their own without intervention.

### Other clinical observations

Body weights and physical examinations were unremarkable throughout the study. The most common abnormal clinical observation/adverse event occurring post-treatment was diarrhea followed by vomiting. However, for diarrhea and vomiting, both the number of abnormal events and the number of animals affected were higher in the control group versus the treated groups. Therefore, neither sign was considered treatment related in this study.

Other non-serious adverse events included melena, eye irritation, ocular discharge, and lameness. All adverse events/abnormal clinical observations were considered unrelated to treatment as they were typical signs for colony dogs, occurred in a single animal, did not occur in a dose dependent manner, and/or occurred in control animals as well. All observations were transient, with only a single animal requiring treatment for an unrelated eye irritation/ocular discharge.

## Discussion

The FDA recommends that labeling for all isoxazoline drugs intended for use in dogs and cats are updated to reflect some of the post-market adverse drug reports regarding neurologic events including muscle tremors, ataxia, and seizures. While the labeling statements may differ slightly between products, the class language states: *Drug “X” (afoxolaner, fluralaner, lotilaner, or sarolaner) is a member of the isoxazoline class. This class has been associated with neurologic adverse reactions including tremors, ataxia, and seizures. Seizures have been reported in dogs receiving isoxazoline class drugs, even in dogs without a history of seizures. Use with caution in dogs with a history of seizures or neurologic disorders.* While the root cause of these neurologic adverse reactions has not been determined, there is speculation that the cause could be related to Pgp or to the isoxazoline not being completely selective for the invertebrate GABA-gated chloride channels. Regardless of the cause, a study in MDR1 mutant dogs helps elucidate any safety finding related to an isoxazoline itself, or the combination of an isoxazoline with other active pharmaceutical ingredients.

As two of the four active ingredients in Credelio Quattro (i.e., lotilaner and moxidectin) are known to have long elimination half-lives [19, 20], the signs of avermectin toxicity may be expected to increase (either in animal rate, incident rate, or both) with repeat dose administrations due to drug accumulation or due to the combination of an isoxazoline with a macrocyclic lactone. Therefore, to provide a more robust evaluation of safety, Credelio Quattro was evaluated at 1×, 2×, and 5× of MRTD following three consecutive monthly administrations. In this study, at the 5× dose level only, the incidence rate of salivation/drooling/vomiting was noted to increase. As mentioned earlier, this finding is not considered clinically relevant in the context of neurological safety as vomiting at higher dose levels is an adverse event experienced in normal healthy dog populations. Therefore, it can be concluded that repeated dosing of Credelio Quattro up to the 5× MRTD in these extremely sensitive dogs did not lead to more frequent or severe toxicity, and only adverse events that are seen in normal healthy dogs (i.e., salivation and vomiting) were observed in this study.

The results of this study can be compared with those of Simparica Trio^®^ (Zoetis) [21], another combination endectocide containing an isoxazoline, a ML anthelmintic, and a pyrimidine-derivative anthelmintic (sarolaner, moxidectin, and pyrantel). Simparica Trio and Credelio Quattro share the same two anthelmintic active ingredients, with the dose of pyrantel being the same in both products. However, the maximum dose of moxidectin in Simparica Trio is 48 µg/kg, which is higher than in Credelio Quattro (40 µg/kg). Simparica Trio was also evaluated in MDR1 mutant dogs at doses of 1×, 3×, and 5× following a once-monthly administration. Simparica Trio was reported to be well tolerated at up to 3× the maximum labeled dose. However, at 5×, a greater number of observations occurred overall and one dog experienced ataxia. While these signs were reported to be mild and self-limiting, they differ from what was observed with Credelio Quattro, where no dogs in the 5× group or any other group experienced ataxia. It is hypothesized that the lower dose of moxidectin in Credelio Quattro contributes to a wider margin of safety observed with Credelio Quattro compared with Simparica Trio in MDR1 mutant dog populations. However, more studies directly comparing the products in the same dogs would be needed to verify the hypothesis.

## Conclusions

This study demonstrated that Credelio Quattro, when administered at 1×, 2×, and 5× the maximum recommended label dose, was well tolerated following three consecutive monthly administrations (28-day intervals) to MDR1 mutant (−/−) dogs. The only treatment-related effect observed in the Collies was salivation/drooling/vomiting, which is a dose-dependent effect that has been reported in normal healthy Beagle dogs and likely does not represent a neurological effect in the Collies. On the basis of the AVS scores combined with the adverse event data, it can be concluded that Credelio Quattro is safe in MDR1 mutant dogs up to five times (5×) the maximum recommended dose.

## Data Availability

Datasets generated and/or analyzed during the current study are not publicly available due to commercial confidentiality of the research. Data not included in the manuscript can only be made available to bona fide researchers subject to a fully executed non-disclosure agreement.

## Abbreviations

ARRIVE: Animal Research Reporting in vitro Experiments
CFR: Code of Federal Regulations
CHK: Cheri-Hill Kennel & Supply, Inc.
FDA: (United States) Food and Drug Administration
GLP: Good Laboratory Practice
LLC: Limited Liability Corporation
ML: Macrocytic lactone
OECD: Organisation for Economic Cooperation and Development
SAS: Statistical analysis system
SOPs: Standard operating procedures
VICH: Veterinary International Conference on Harmonization
MDR1: multi-drug resistance 1
MRTD: maximum recommended therapeutic dose
Pgp: P-glycoprotein
